# A framework for the development and appraisal of diet and eating behaviour outcome measures

**DOI:** 10.1186/1745-6215-14-S1-P71

**Published:** 2013-11-29

**Authors:** Dianne Ward, Maria Bryant, Claudia Gorecki, Amber Vaughn, Rachel Tabak, June Stevens, Jane Nixon, Susan Jebb, Kath Roberts, Judy Wright, Julia Brown

**Affiliations:** 1 University of Leeds, Leeds, UK; 2 University of North Carolina, Chapel Hill, NC, USA; 3 University in St. Louis,, St. Louis, MO, USA; 4 MRC Human Nutrition Research, Cambridge, UK; 5 National Obesity Observatory, Oxford, UK

## Background

Multiple existing tools provide guidance for developing and evaluating patient reported outcome measures (e.g. Scientific Advisory Committee (SAC) of the Medical Outcomes Trust guidelines, FDA guidance for Patient Reported Outcomes). Guidance is absent, however, for diet and eating behaviour outcome measures that can be used to evaluate community-based interventions.

## Aim

To develop a framework to guide the development and appraisal of behavioural outcome measures of diet and eating behaviours.

## Methods

An appraisal framework, based on relevant items from existing tools (i.e. COSMIN, FDA, EMPRO, SAC) was developed. Initial testing was conducted as part of a systematic review, in which data were extracted from 100 papers describing parental feeding practices measures (HomeSTEAD). Next, the framework was expanded to include a scoring system for items related to conceptual framework, reliability, validity and responsiveness testing. The expanded framework was then used in a systematic review of 353 papers describing outcome measures used in childhood obesity treatment evaluations (CoOR).

## Results

An appraisal framework guided the development of extraction forms for diet and eating behaviour outcome measures. A key part of the framework was a scoring system, which enables individual outcome measures to be scored (1-4) to appraise measurement development (description of concept, use of population); reliability (internal consistency, test re-test, inter-rater); validity (factor analysis, criterion, convergent, construct); and responsiveness.

## Conclusions

This work provides a framework to enable researchers to appraise existing outcome measures for use in behavioural intervention trials and can guide development of new diet and eating behaviours outcome measures.

